# A fast pace-of-life is traded off against a high thermal performance

**DOI:** 10.1098/rspb.2021.2414

**Published:** 2022-04-13

**Authors:** Nedim Tüzün, Robby Stoks

**Affiliations:** Laboratory of Evolutionary Stress Ecology and Ecotoxicology, KU Leuven, Charles Deberiotstraat 32, 3000 Leuven, Belgium

**Keywords:** trade-off, life-history, thermal physiology, syndromes, structural equation modelling, Odonata

## Abstract

The integration of life-history, behavioural and physiological traits into a ‘pace-of-life syndrome’ is a powerful concept in understanding trait variation in nature. Yet, mechanisms maintaining variation in ‘pace-of-life’ are not well understood. We tested whether decreased thermal performance is an energetic cost of a faster pace-of-life. We characterized the pace-of-life of larvae of the damselfly *Ischnura elegans* from high-latitude and low-latitude regions when reared at 20°C or 24°C in a common-garden experiment, and estimated thermal performance curves for a set of behavioural, physiological and performance traits. Our results confirm a faster pace-of-life (i.e. faster growth and metabolic rate, more active and bold behaviour) in the low-latitude and in warm-reared larvae, and reveal increased maximum performance, *R*_max_, but not thermal optimum *T*_opt_, in low-latitude larvae. Besides a clear pace-of-life syndrome integration at the individual level, larvae also aligned along a ‘cold–hot’ axis. Importantly, a faster pace-of-life correlated negatively with a high thermal performance (i.e. higher *T*_opt_ for swimming speed, metabolic rate, activity and boldness), which was consistent across latitudes and rearing temperatures. This trade-off, potentially driven by the energetically costly maintenance of a fast pace-of-life, may be an alternative mechanism contributing to the maintenance of variation in pace-of-life within populations.

## 1. Introduction

Many organisms exhibit correlated suites of life-history, behavioural and physiological traits forming a pace-of-life syndrome [1,2]. Individuals with a fast pace-of-life typically grow and develop faster, show more active, bold and aggressive personalities, have a higher metabolic rate, reproduce earlier, and live shorter [1–3]. While proven to be a very useful concept in understanding trait (co)variation in nature, the maintenance of pace-of-life types within populations is still debated [4]. Well-studied trade-offs such as growth versus survival and current versus future reproduction have been suggested as underlying mechanisms of these adaptive trait integration patterns [4,5], hence allowing for the co-existence of individuals with contrasting pace-of-life within a population. Only very recently have researchers started to investigate alternative mechanisms that may explain intra-population variation in the pace-of-life syndrome [6]. Energy-mediated trade-off patterns with tolerance to environmental stressors, such as increasing temperatures [7], are promising candidates in this context, given stress coping mechanisms are energy-demanding [8].

An emerging insight is that organisms can be aligned across a ‘cold–hot’ axis: individuals situated on the ‘hot’ end of this axis are said to have a higher thermal performance, i.e. prefer higher environmental temperatures and show a higher maximum performance at higher temperatures, compared to ‘cold’ individuals (e.g. [9,10]). This axis can be described by reconstructing individual thermal performance curves, describing the relationship between a trait and a temperature gradient [11]. Typically, thermal performance curves have an accelerating rising part (activation energy, *E*_a_) until reaching an optimum temperature (*T*_opt_) at which maximum performance (*R*_max_) is achieved, followed by a fast decelerating part until the critical maximum temperature where performance is zero [11,12]. Individual variation in thermal performance curves reflects differences in the ability to cope with thermal regimes [11,13]. Specifically, the frequently documented individual-level positive correlation between *T*_opt_ and *R*_max_ (e.g. [14]) reflects increased performance at higher temperatures, the so-called ‘hotter-is-better’ hypothesis [12]. Trade-off patterns between a single thermal performance curve parameter and the trait values of a single pace-of-life trait have occasionally been shown. Specifically, a trade-off at the individual level has been detected between a high *T*_opt_ for locomotor performance and a fast growth rate [15,16]. Yet, such trade-off patterns have not been considered between integrated sets of (i) multiple thermal performance curve parameters of multiple pace-of-life-related traits (i.e. ‘cold–hot’ axis) and (ii) their trait values (i.e. pace-of-life syndrome). This gap in the literature is important because organismal traits form integrated sets [17], yet their thermal performance curves can be strongly different [11,18]. Furthermore, because environmental conditions can shape trade-off patterns [19], the relationship between pace-of-life traits [7,20] and between thermal performance curve parameters of the same trait [14,21] may depend on the evolutionary background of populations and on experimental treatments (but see: [22]). Such conditional trait integration patterns may also apply to the relationship between the pace-of-life syndrome and thermal performance, yet this has never been tested.

We here tested for alignment at the individual level across a slow–fast (pace-of-life) axis and along a cold–hot (thermal performance) axis, to then evaluate a trade-off relationship between the pace-of-life and thermal performance. We additionally studied whether these among-individual trait integration and trade-off patterns were robust across two latitudes and two rearing temperatures. For this, we conducted a full-factorial common-garden experiment with larvae of the damselfly *Ischnura elegans* originating from high-latitude (Denmark and Southern Sweden) and low-latitude (Southern France) populations being reared at 20°C or 24°C. We characterized for each larva the pace-of-life by measuring a set of behavioural, life-history and physiological traits, and the thermal performance by estimating thermal performance curves and associated parameters (*E*_a_, *T*_opt_ and *R*_max_) for the same set of behavioural and physiological traits, and for a locomotor performance trait. We explicitly tested at the individual level for pace-of-life and thermal performance patterns, and for a trade-off between these using a structural equation modelling (SEM) framework.

We had predictions both at the level of the latitudes and temperature treatments, and at the individual level. First, based on the higher number of generations per year in the low- versus high-latitude populations (three to four generations per year versus one generation per 2 years: [23]), we expected a faster pace-of-life in the more time-constrained low-latitude populations, as documented in the study species [22,24]. We also expected a faster pace-of-life in larvae when reared at the warmer versus colder temperature, based on the metabolic theory of ecology [25] (e.g. for the water flea *Daphnia*: [7]). Regarding the thermal performance curves, we expected an overall higher performance, including a higher *R*_max_, in low-latitude larvae to cope with the stronger time constraints [26], but not necessarily a higher *T*_opt,_ given the difficulty of imposing radical changes to the structural and physiological characteristics of evolutionarily conserved enzymes [12]. Nevertheless, a higher *T*_opt_ in populations originating from warmer latitudes has also been reported and is interpreted as thermal adaptation to local thermal conditions [27,28]. Second, we expected at the individual level the existence of a pace-of-life syndrome as previously shown in the study species [22,29]. Based on intensive work on lizards [9,10], we expected positive covariations between thermal performance curve parameters (particularly *T*_opt_ and *R*_max_) of a given trait, as well as across different traits, to form a ‘cold–hot’ axis (e.g. ‘hot’ individuals have higher *T*_opt_ and *R*_max_ for all traits). Finally, given the associated costs of sustaining a fast pace-of-life [1] and a hot thermal performance [13], we expected a negative correlation between the pace-of-life and thermal performance. Hence, we predicted fast-paced larvae to be situated more on the cold end of the cold-–hot axis, and vice versa for slow-paced larvae. On the other hand, in case a ‘hot’ thermal performance or a fast pace-of-life do not carry significant costs, a positive correlation between the pace-of-life and thermal performance may also be expected, as individuals with a proactive behaviour (more aggressive, bold and active) can potentially compensate for the increased metabolic demands at higher body temperatures [9,10].

## 2. Material and methods

We studied three low-latitude (southern France) and three high-latitude (Denmark and southern Sweden) populations at shallow lakes within the European range of *I. elegans* [30] (coordinates in the electronic supplementary material, table S1). We collected egg clutches of *ca* 10 females per population. We reared larvae individually in 200 ml plastic vials filled with dechlorinated tap water that were placed in incubators at either 20 or 24°C at a photoperiod of 14 : 10 light : dark. Rearing temperatures reflect mean summer water temperatures of shallow lakes in the study region (southern Sweden *ca* 20°C, southern France *ca* 24°C: [22,31]). Larvae were fed *ad libitum* nauplii of *Artemia* six times a week.

As a general research strategy, we conducted two consecutive sets of trait measurements with distinct aims (electronic supplementary material, figure S1). First, to characterize the pace-of-life of each larva, we estimated larval growth rate, activity and boldness behaviour, and metabolic rate at their rearing temperature, in that order. Next, to characterize the thermal performance of each larva, we reconstructed thermal performance curves for the same behavioural (activity and boldness) and physiological (metabolic rate) traits, as well as for swimming speed, at each of six test temperatures (16, 20, 24, 28, 32 and 36°C) after a 1 h acclimatisation period. For thermal performance curves, we ran trials at two temperatures per day and conducted trials every other day. We randomized the order of the six test temperatures, except for the highest two temperatures (32 and 36°C), which were conducted on the final trial day to prevent potential carry-over effects of exposure to extreme high temperatures (following: [32,33]). We measured growth rate as gain in body mass over 4 days, activity as total distance moved in 10 min, boldness as latency time to resume activity following a simulated predator attack, metabolic rate as the rate of oxygen consumption, and swimming speed as the speed of the fastest escape swimming bout. Detailed protocols of trait measurements are provided as electronic supplementary material. Sample sizes per treatment combination were 38 high-latitude larvae at rearing temperature 20°C, 53 high-latitude larvae at 24°C, 55 low-latitude larvae at 20°C and 43 low-latitude larvae at 24°C. For the thermal performance curve reconstructions, this resulted in 1134 measurements for each of four response variables.

### (a) Statistical analyses

To test for effects of rearing temperature and latitude on growth rate, activity, boldness and metabolic rate when measured at their rearing temperature (prior to the thermal performance curve trials), we ran separate linear mixed-effect models (LMMs) with temperature, latitude and their interaction term as fixed effects, and population (nested within latitude) as a random effect. Full statistical results are provided in the electronic supplementary material, table S2. To test for effects of rearing temperature and latitude on thermal performance curves of activity, boldness, metabolic rate and swimming speed, we ran separate LMMs as described above, with the addition of the linear and quadratic terms of test temperature, as well as their interactions with rearing temperature and latitude. To prevent over-parameterization of these quadratic models, we applied stepwise backwards elimination model selection using the *step* function in the software R. For each trait, we estimated the repeatability [34] across test temperatures (as in [35]), adjusted for fixed effects rearing temperature and latitude (see electronic supplementary material for details of repeatability models).

To test for treatment effects on the extracted thermal performance curve parameters (*R*_max_, *T*_opt_ and *E*_a_, see below), we ran separate LMMs with temperature, latitude and their interaction term as fixed effects, and population (nested within latitude) as a random effect. As the random effect of population was never significant (likelihood ratio tests, *p* > 0.446 for all models described above), we removed this factor from the models. Detailed results of these models (electronic supplementary material, table S3) as well as estimates (with 95% CIs) of thermal performance curve parameters (electronic supplementary material, table S4) are reported as electronic supplementary material.

#### (i) Estimation of thermal performance curves and their parameters

Following a recently published pipeline for analysing thermal performance curves [36], we fitted a set of candidate thermal performance curve models separately for activity, boldness, metabolic rate and swimming speed, and this for each of the four combinations of rearing temperature and latitude. Based on AIC comparisons, the quadratic thermal performance curve model was found to be the best overall fitting model (details provided in electronic supplementary material). Next, we fitted the quadratic function for each larva separately and extracted three thermal performance curve parameters per trait that are known to have biological and ecological relevance [12]: maximum performance (*R*_max_), thermal optimum (*T*_opt_, the temperature at which maximum performance is achieved) and activation energy (*E*_a_, the slope of the increasing linear part of thermal performance curves).

#### (ii) Structural equation modelling

**Figure 1.**
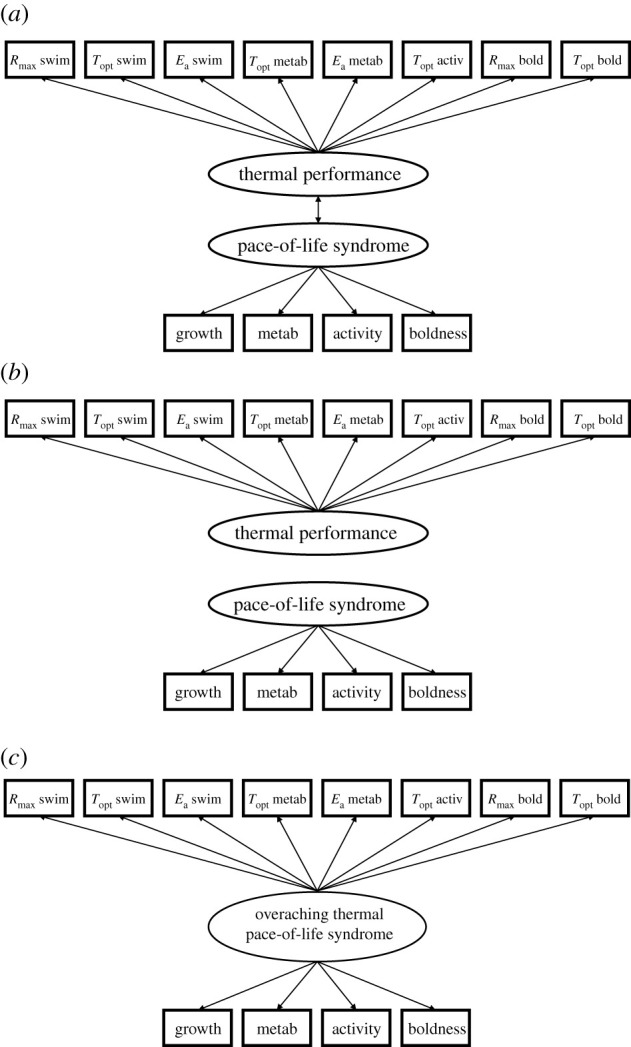
Diagrams of the three *a priori* candidate structural equation models depicting different phenotypic covariance structures between the pace-of-life and thermal performance: (*a*) ‘coupled pace-of-life syndrome–thermal performance’ model, (*b*) ‘independent pace-of-life syndrome and thermal performance’ model and (*c*) ‘overarching thermal pace-of-life syndrome' (*c*). Observed variables are given in rectangles, unmeasured latent variables in ellipses. See main text for details.

To test the hypotheses that (i) pace-of-life traits integrate to form a slow–fast axis, (ii) thermal performance curve parameters (within and across traits) integrate to form a cold–hot axis and (iii) the slow–fast and cold–hot axes are correlated, we used structural equation modelling (SEM) [37]. SEM is often used in ‘syndrome’ studies (e.g. [38]), as it allows to construct latent variables that reflect integrated traits sets (here the pace-of-life syndrome and cold–hot axis), and to test for relationships between these latent variables. For this, we constructed SEMs with two latent variables that were hypothesized to separately represent the pace-of-life syndrome and the thermal performance of the larvae (figure 1). The pace-of-life syndrome latent variable comprised four indicator (observed) variables (growth rate, activity, boldness and metabolic rate) that were measured at the rearing temperature (20°C or 24°C) of a larva, prior to the thermal performance curve trials. To refine the models, we excluded several thermal performance curve parameters that were shown to have relatively low predictive power (see electronic supplementary material) and included (residual) correlations between the thermal performance curve parameters and between the pace-of-life traits, as recommended by modification indices (i.e. indices that estimate the amount by which the model *χ*^2^ would be reduced if a single parameter restriction were to be removed from the model).

As a first step, to test the null hypothesis that assumes no trait integration of pace-of-life traits (slow–fast axis) or thermal performance curve parameters (cold–hot axis), we constructed a model with no latent variables. This model was rejected, as indicated by the significant deviation between observed and model-implied covariances (*χ*^2^ = 135.8, d.f. = 48, *p* < 0.001), hence was disregarded. Next, to test whether the pace-of-life syndrome and thermal performance were linked, we constructed two models that use the latent structures as described before, whereby one model included a correlation between the two latent variables (‘coupled pace-of-life syndrome–thermal performance’ model, figure 1*a*), while the other model did not include this correlation (‘independent pace-of-life syndrome and thermal performance’ model; figure 1*b*). As a third alternative hypothesis, we also constructed a non-hierarchical, ‘overarching thermal pace-of-life syndrome' model (figure 1*c*), whereby thermal performance curve parameters and pace-of-life traits were tested for their integration into a single latent variable (as in: [39]). To determine the best-fitting model, we compared the AIC scores of the three models and further confirmed the fit of the best model (i.e. model with lowest AIC score with ΔAIC > 2) by assessing the fit indices typically used in SEM. Following index values are indicative of models that fit the data well [40]: *p* > 0.05 for a *χ*^2^-test, a root-mean-square error of approximation (RMSEA) ≤ 0.05, a standard root-mean-square residual (SRMR) ≤ 0.08 and a comparative fit index (CFI) ≥ 0.95.

Finally, to test whether the integration of pace-of-life syndrome and thermal performance traits depended on the rearing temperature and latitude of origin (or their interaction), we conducted a multi-group SEM using the best-fitting model among the three SEMs (described above) (as in: [20,22]). We used rearing temperature (two levels), latitude (two levels) or the composite variable of rearing temperature × latitude (four levels) as a grouping variable in separate models, and AIC-compared models where the factor loadings on latent variables and correlation between the two latent variables were either the same (constrained model), or allowed to vary between the groups, i.e. treatments (free model). A better fit of the free model would indicate treatment-dependent trait integration patterns.

All analyses were conducted in R v. 4.0.5 [41], using the packages *lme4* for LMMs [42], *emmeans* for post hoc tests [43] and *lavaan* for SEM [44]. We used the packages *rTPC* and *nls.multstart* [36] to fit thermal performance curve models and to calculate thermal performance curve parameters. To conform with model assumptions, we square root-transformed activity and log-transformed boldness.

## 3. Results

### (a) Effects of latitude and rearing temperature on trait means

**Figure 2.**
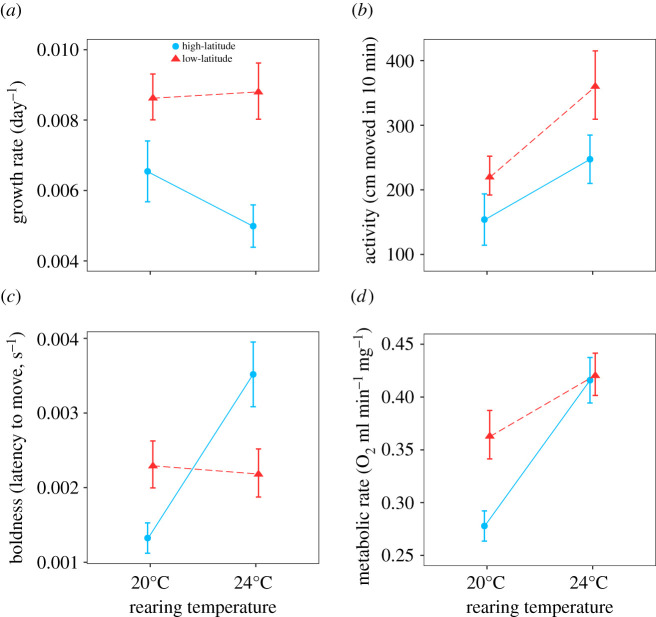
Effects of latitude and rearing temperature on the trait means (±1 s.e.) for (*a*) growth rate, (*b*) activity, (*c*) boldness and (*d*) metabolic rate. (Online version in colour.)

Growth rates were higher for low-latitude compared to high-latitude larvae (figure 2*a*; electronic supplementary material, table S2). Rearing temperature and its interaction with latitude did not affect growth rates. Activity was higher for the low-latitude larvae, and for larvae developed at the higher rearing temperature of 24°C (figure 2*b*). For boldness behaviour, the significant latitude-by-rearing temperature effect indicated that the higher rearing temperature resulted in increased boldness in the high-latitude (contrast test: *p* < 0.001) but not in the low-latitude larvae (*p* = 0.810) (figure 2*c*). Metabolic rates were higher for larvae that developed at 24°C compared to 20°C, whereas low-latitude larvae had higher metabolic rates than high-latitude larvae when developed at 20°C (*p* = 0.005), but not at 24°C (*p* = 0.848) (*p* = 0.055; latitude × rearing temperature, figure 2*d*).

### (b) Effects of latitude and rearing temperature on thermal performance curves

**Figure 3.**
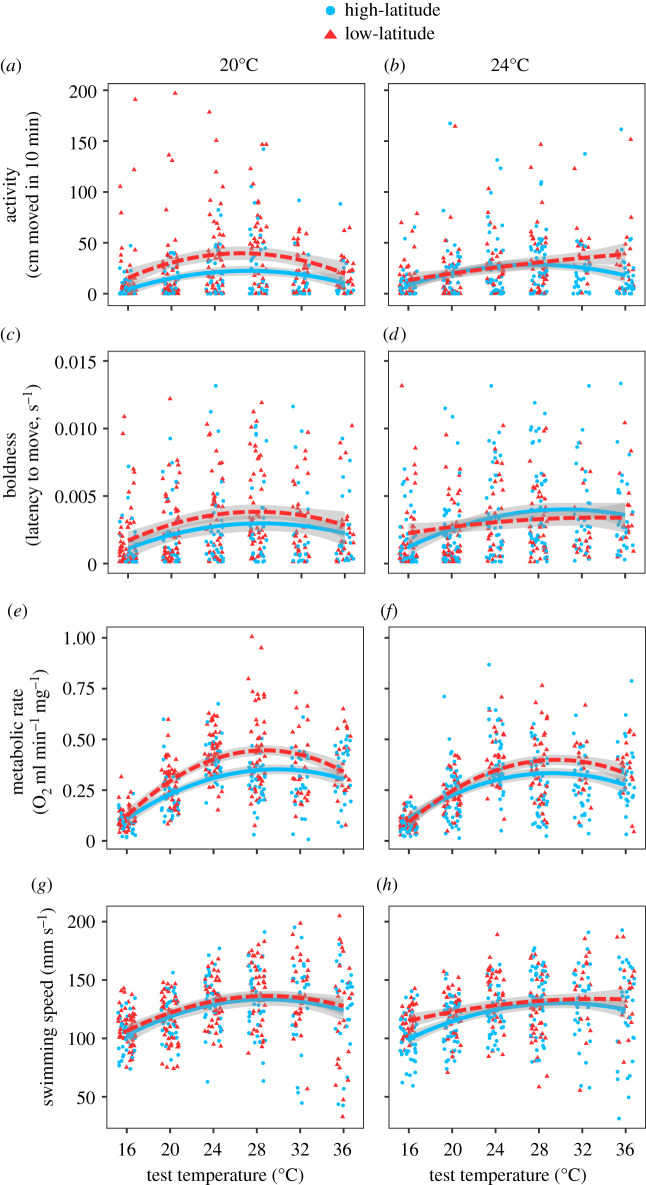
Thermal performance curves of (*a*,*b*) activity, (*c*,*d*) boldness, (*e*,*f*) metabolic rate and (*g*,*h*) swimming speed, as a function of rearing temperatures and latitudes. Solid (blue) curves are for high-latitude larvae; dashed (red) curves are for low-latitude larvae. Bands around curves represent 95% CIs. (Online version in colour.)

Adjusted repeatabilities calculated across test temperatures were as following: *R* [95% CI] = 0.22 [0.15, 0.29] for activity, 0.24 [0.17, 0.31] for boldness, 0.15 [0.08, 0.22] for metabolic rate, and 0.17 [0.11, 0.24] for swimming speed. For all traits, the thermal performance curves had a quadratic component with typically a rising part up to an optimum, followed by a decreasing part (figure 3). This was further indicated by the significant effects of the linear (‘t_temp’) and quadratic terms (‘t_temp^2^’) of test temperature for all traits (table 1). Low-latitude larvae had overall higher activity and boldness values than high-latitude larvae across test temperatures when reared at 20°C temperature (contrast test: activity, *p* < 0.001; boldness, *p* = 0.001), but not at 24°C (activity, *p* = 0.709; boldness, *p* = 0.575) (latitude × rearing temperature, table 1 and figure 3*a*–*d*). This pattern was further reflected in low-latitude larvae having higher maximum performance (*R*_max_) than high-latitude larvae when developed at 20°C but not at 24°C (latitude × rearing temperature, electronic supplementary material, tables S3 and S4). The shape of the thermal performance curve for activity was influenced by the latitude and rearing temperature (test temperature^2^ × latitude × rearing temperature, table 1): the quadratic term of test temperature was significant for three of the four treatment combinations (all *p* < 0.001), except for low-latitude larvae reared at 24°C (*p* = 0.803), where the thermal performance curve was mainly linear but with a less steep increase at higher temperatures (figure 3*a,b*). This was partly mirrored in the slightly higher activation energy (*E*_a_) for activity in low- compared to high-latitude larvae (trend), and in larvae reared at 24°C compared to 20°C (electronic supplementary material, table S4, but no significant latitude × rearing temperature interaction for *E*_a_, electronic supplementary material, table S3). The thermal optimum (*T*_opt_) for activity was not significantly influenced by the treatments (electronic supplementary material, table S3). For boldness, the effects of latitude and rearing temperature on the thermal performance curve were similar to for activity, but did not reach significance (table 1 and figure 3*c,d*); except for the *T*_opt_ being slightly higher in high-latitude larvae (electronic supplementary material, tables S3 and S4).

**Table 1.** Results of linear models testing for treatment effects on thermal performance curves. t_temp = test temperature (t_temp^2^ = quadratic term of test temperature), lat = latitude, r_temp = rearing temperature. Significant effects (*p* < 0.05) are indicated in italics.

	activity			boldness			metabolic rate			swimming speed		
	d.f.	*F*	*p*	d.f.	*F*	*p*	d.f.	*F*	*P*	d.f.	*F*	*p*
test temperature	*1,870*	*32.08*	*<0.001*	*1,862*	*64.78*	*<0.001*	*1,886*	*306.80*	*<0.001*	*1,905*	*90.65*	*<0.001*
test temperature^2^	*1,870*	*40.64*	*<0.001*	*1,862*	*28.03*	*<0.001*	*1,886*	*201.58*	*<0.001*	*1,905*	*47.64*	*<0.001*
latitude	*1,870*	*29.85*	*<0.001*	*1,862*	*9.87*	*0.002*	*1,886*	*39.53*	*<0.001*	*1,905*	*12.02*	*0.001*
rearing temperature	1,870	1.73	0.189	*1,862*	*10.29*	*0.001*	*1,886*	*9.10*	*0.003*	1,905	0.25	0.619
lat × r_temp	*1,870*	*7.45*	*0.007*	*1,862*	*5.52*	*0.019*	1,886	2.59	0.108			
t_temp × lat	1,870	2.47	0.117	1,862	2.21	0.137	*1,886*	*5.64*	*0.018*			
t_temp × r_temp	1,870	3.13	0.077	1,862	2.51	0.114				1,905	0.04	0.836
t_temp × lat × r_temp	1,870	0.60	0.438	1,862	1.96	0.162						
t_temp^2^ × lat	1,870	1.17	0.280	1,862	0.17	0.676	*1,886*	*6.73*	*0.010*			
t_temp^2^ × r_temp	*1,870*	*3.90*	*0.049*	1,862	2.09	0.149				1,905	2.35	0.125
t_temp^2^ × lat × r_temp	*1,870*	*3.86*	*0.050*	1,862	2.26	0.133						

Metabolic rates were overall higher for low- compared to high-latitude larvae, and slightly higher for larvae developed at 20°C compared to 24°C across the test temperatures (table 1 and figure 3*e,f*). Higher *R*_max_ values for low-latitude larvae (electronic supplementary material, table S4) are in line with this pattern, but no significant effect of rearing temperature on *R*_max_ was detected (electronic supplementary material, table S3). Both the linear increase and the quadratic shape of the thermal performance curve for metabolic rate was affected by the latitude (test temperature × latitude and test temperature^2^ × latitude, table 1 and figure 3*e,f*). The linear increasing part of the curve was steeper in low-latitude larvae (estimate ± s.e. = 0.107 ± 0.008) compared to high-latitude larvae (0.075 ± 0.008). This pattern was partially confirmed by the finding of a slightly higher *E*_a_ (trend) in the low-latitude larvae (electronic supplementary material, tables S3 and S4). Moreover, the thermal performance curve was more concave-shaped, i.e. the quadratic temperature estimate was stronger, for low-latitude larvae (estimate ± s.e. = −0.0018 ± 0.0001) compared to high-latitude larvae (−0.0013 ± 0.0001).

Swimming speeds were overall higher for the low-latitude larvae across the test temperatures (table 1 and figure 3*g*,*h*). Mean swimming speed values, as well as the magnitude of the linear increase and shape of the thermal performance curve, did not depend on any of the treatments (table 1 and figure 3*g*,*h*), although the *T*_opt_ was slightly higher in high-latitude larvae (electronic supplementary material, tables S3 and S4). Using mass-corrected swimming speeds yielded virtually identical results (not shown).

### (c) Trait covariation patterns at the individual level

**Figure 4.**
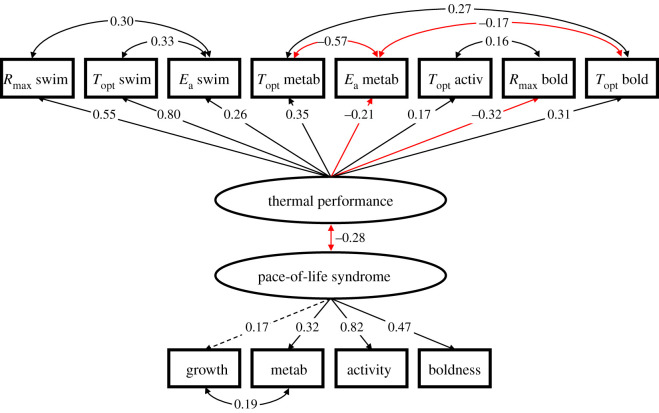
SEM diagram depicting the supported ‘coupled pace-of-life syndrome–thermal performance’ model. Numbers associated with single-headed arrows are standardized path coefficients. Double-headed arrows indicate correlations. All loadings were significant (*p* < 0.05) with the exception of growth rate (dotted arrow, *p* = 0.068). Red arrows indicate negative path coefficients; black arrows indicate positive path coefficients. (Online version in colour.)

Comparisons of candidate SEMs revealed the ‘coupled pace-of-life syndrome–thermal performance model’ as the best fit for the data (electronic supplementary material, table S7). Note that the other two candidate models were rejected (*p* ≤ 0.05, electronic supplementary material, table S7). Rejection of the ‘overarching thermal pace-of-life syndrome' model (see figure 1*c*) indicated that the set of pace-of-life traits and the set of thermal performance traits did not integrate into a single latent variable. This best model (figure 4) indicated (i) the presence of a pace-of-life syndrome latent variable, comprising of growth rate, activity, boldness and metabolic rate (measured at the rearing temperature), (ii) the presence of a thermal performance latent variable, comprising of *R*_max_ (for swimming speed and boldness), *T*_opt_ (for swimming speed, metabolic rate, activity and boldness) and *E*_a_ (for swimming speed and metabolic rate) and (iii) an integration of the two latent variables. The pace-of-life syndrome latent variable indicated that more active larvae were also bolder and had higher metabolic rates and faster growth rates (trend). The two behaviours were the strongest contributors (i.e. had the highest loadings) to the pace-of-life syndrome. As for the thermal performance latent variable, the positive loadings of *T*_opt_ for all traits indicated that larvae with higher *T*_opt_ values for one trait also had higher *T*_opt_ values for the other measured traits. Moreover, larvae with higher *T*_opt_ values also had a higher *R*_max_ and *E*_a_ for swimming speed but lower values of *R*_max_ for boldness and of *E*_a_ for metabolic rate. The two strongest contributors to thermal performance were *R*_max_ and *T*_opt_ for swimming speed (both with positive loadings). Notably, the two latent variables were negatively correlated, indicating larvae with a faster pace-of-life had lower values for thermal performance (e.g. lower *T*_opt_ values).

A multi-group SEM approach using AIC scores revealed that the ‘constrained’ models, i.e. models in which loadings and correlations were fixed across treatments, to have the best fit (see electronic supplementary material, table S8).

## 4. Discussion

Next to the findings of a trait integration of life-history, physiology and behavioural traits into a pace-of-life syndrome (indicating a ‘slow–fast’ axis), and a trait integration of thermal performance traits (indicating a ‘cold–hot’ axis), a major finding of our study was the presence of a trade-off pattern where animals with a faster pace-of-life showed a lower thermal performance. To provide background, we first discuss latitudinal and thermal patterns in trait means and thermal performance curves, and then discuss this trade-off.

### (a) Pace-of-life across latitudes and rearing temperatures

As expected, low-latitude larvae showed a faster pace-of-life (faster growth and metabolic rate, and higher activity) compared to high-latitude larvae ([1,45], for the study species: [22]). The higher number of generations produced per year in the low-latitude populations is assumed to be the driver of their faster pace-of-life. Producing multiple generations in a given year requires a fast life-history (i.e. growth rate) which often requires a fast metabolic machinery (i.e. metabolic rate) and a behavioural profile that facilitates increased energy acquisition (i.e. activity).

We also confirmed previous findings showing an accelerated pace-of-life (for metabolic rate and activity, and for boldness in high-latitude populations) when reared at a higher temperature (e.g. in the water flea *Daphnia magna*: [7]). This thermally induced faster pace-of-life was more prominent in high-latitude compared to low-latitude larvae (for metabolic rate and boldness), suggesting a potential compensatory mechanism whereby high-latitude populations more efficiently capitalize on higher temperatures, which they encounter less frequently (see [46] for a similar reasoning in the cooler high-elevation populations of a grasshopper).

### (b) Thermal performance curves across latitudes and rearing temperatures

Previous work documenting increased *R*_max_ in low-latitude populations (e.g. the common woodlouse *Porcellio laevis*: [27]; the porcelain crab *Petrolisthes violaceus*: [28]) appears in line with our findings. Yet, these studies also reported higher *T*_opt_ in low-latitude populations, in line with the ‘hotter-is-better’ hypothesis that predicts thermal adaptation to facilitate increased maximum performance at higher temperatures in warm-adapted populations [12,47]. Instead, we observed a higher *R*_max_ in the absence of a change in *T*_opt_ in the more time-constrained low-latitude populations. This resembles ‘countergradient variation’ [26], whereby populations experiencing stronger time constraints show higher (maximum) growth and development rates to compensate for a short growing season. Countergradient variation has, for example, been documented in fish along a latitudinal gradient [48], and in damselflies along an urbanization gradient [49]. The higher maximum performance in the more time-constrained low-latitude populations for activity, boldness and metabolic rate (traits that are tightly linked to an accelerated life history) is in line with this pattern.

The mostly similar *T*_opt_ values across the latitudes indicate no evidence for a thermal adaptation scenario [12]. While higher *T*_opt_ in the warmer lower latitude populations has been shown in other ectotherms (e.g. [27,28]), the absence of differentiation in *T*_opt_ across geographically separated populations is not uncommon (e.g. [50,51]) (see also [52] for a review of geographic variation in insect thermal performance curves). Radical changes in the thermokinetics of enzymes, e.g. an increase in temperatures at which an enzyme can function, may require fundamental changes in the genetic architecture, such as mutations in structural genes [12]. Thermokinetics of enzymes are therefore assumed to be evolutionarily conserved systems, which may partially explain findings of relatively stable *T*_opt_ across latitudes.

### (c) Pace-of-life syndrome, cold–hot axis, and their link

In accordance with the pace-of-life syndrome hypothesis [1,2], life-history, physiology and behavioural traits showed a positive covariation at the individual level, forming a ‘slow–fast’ axis. Specifically, larvae at the fast end of the axis had a higher growth rate, were more active and bolder, and showed a faster metabolic rate. Behaviours that have direct links to energy acquisition, e.g. activity and boldness (for damselflies: [53]), are expected to positively covary with metabolic rate and life history [54,55]. Note that the relatively low trait repeatabilities (all less than 0.25) calculated across the six test temperatures are likely underestimations of the more traditional, across-time repeatabilities, calculated at different time points but at the same temperature. We indeed have observed substantially higher across-time repeatabilities for the study species and for a closely related damselfly species [29,56]. The previous finding of pace-of-life syndrome with a similar trait integration pattern [22] further suggests a consistent pace-of-life syndrome in the study system.

Next to the slow–fast axis, the tight integration of thermal performance traits at the individual level confirmed the existence of the recently proposed ‘cold–hot’ axis, that had only been studied in lizards [9,10]. By including thermal performance curve parameters of activity, boldness and metabolic rate, we extended this cold–hot axis, which in previous studies was based on locomotor performance and thermal preference only. Animals at the hot end of the axis showed a higher thermal performance as evidenced by the consistently higher *T*_opt_ values for swimming speed, metabolic rate, activity and boldness, and also a higher *R*_max_ for swimming speed, the trait that contributed most to the cold–hot axis. The positive covariation of *T*_opt_ across traits (see also [11,18]) occurred despite the different thermal performance curves, hence also *T*_opt_ among traits. The positive association at the individual level between *R*_max_ and *T*_opt_ for swimming performance fits the ‘hotter-is-better’ hypothesis [12] and has been reported previously for locomotor performance (in delicate skinks: [9,10]; in the European common frog: [14]). This pattern was not observed for other traits (metabolic rate and activity) and was even reversed for boldness, suggesting that the thermodynamic constraints assumed to underlie the hotter-is-better hypothesis can be trait dependent.

A key novelty was a coupling of the ‘slow–fast’ axis and the ‘cold-–hot’ axis, specifically a trade-off pattern where animals with a faster pace-of-life showed a lower thermal performance. Indeed, animals with a faster pace-of-life were situated on the ‘colder’ side along the cold–hot continuum, i.e. had a lower *T*_opt_ for all four traits, and a lower *R*_max_ for swimming speed. The two studies that tested a relationship between (non-behavioural) pace-of-life traits and thermal performance traits only considered locomotor traits, and also reported a negative correlation between growth rate and *T*_opt_ (tadpoles: [15]; lizards: [16]). Sustaining a faster pace-of-life is assumed to be costly [1] and therefore may require allocation of energy away from costly processes related to thermal performance [13]. The biochemical machinery allowing maximum performance at higher temperatures may indeed be traded off with a fast pace-of-life. For example, an increase in maximum thermal tolerance, a thermal performance parameter often shown to positively covary and coevolve with *T*_opt_ [57], has been shown to trade-off with growth rate (e.g. in the rainbow trout: [58]). Another potential mechanism underlying a high *T*_opt_, namely the costly production of heat shock proteins to prevent protein denaturation at high temperatures [59], has also been shown to trade-off with growth rate [60] (for the study species: [61]). Whatever the exact underpinnings of the negative coupling between the pace-of-life syndrome and the cold–hot axis, this trade-off pattern may be an ignored, but potentially important mechanism for the co-existence of individuals with contrasting pace-of-life within a population. The mechanisms maintaining variation of the pace-of-life within a population are still highly debated [1,4] and are typically based on trade-offs between pace-of-life traits; i.e. growth versus survival [5], or current versus future reproduction [4]. Our data suggest that another reason why fast-paced individuals may not always be favoured is that they have a lower thermal performance.

Intriguingly, the two other studies that explicitly looked at the link between behavioural pace-of-life traits and thermal performance, both on the terrestrial skink *Lampropholis delicata*, found instead a positive covariation whereby animals showing higher exploration, activity and boldness had also a higher *T*_opt_ and *R*_max_ for sprint speed, and a higher thermal preference [9,10]. These authors therefore suggested to extend the traditional pace-of-life syndrome to include thermal performance. The absence of a trade-off between fast pace-of-life and thermal performance may point to reduced costs of having a ‘hot’ thermal type in this system. Potentially, because lizards are commonly exposed to strongly fluctuating, hence also very high temperatures, they may be energetically adapted to develop ‘hotter’ thermal types with reduced energy allocation. It is important to note that such a positive association between behaviour and thermal performance may indeed be expected for terrestrial, heliothermic animals that rely heavily on behavioural thermoregulation such as basking behaviour, as lizards with a ‘hot’ type that also have a more proactive behavioural type (i.e. more active, exploratory and bold) can generate more basking opportunities. Given that aquatic insects such as damselfly larvae are better protected from drastic temperature fluctuations (both in time and space) due to the buffering effect of water, a positive relationship between pace-of-life and thermal performance driven by thermoregulatory behaviour is much less expected. A recent study indeed reported no link between preferred temperature and behavioural type, i.e. bold versus shy, in a freshwater fish [62] (but see [63]).

Another key finding was that the covariation patterns, both among pace-of-life traits and among thermal performance traits (i.e. thermal performance curve parameters) as well as the trade-off pattern between pace-of-life and thermal performance, were stable across latitudes and rearing temperatures. Consistency in the structure of pace-of-life syndromes (see also: [22]) and thermal performance traits may impose restrictions on independent (evolutionary) responses of individual traits to warming, assuming the syndrome structure is underpinned by genetic correlations [64]. Yet, note that this may not necessarily constrain adaptive evolution of the integrated trait sets (i.e. the pace-of-life syndrome and thermal performance), given that adaptive trait integration may potentially guide adaptive evolution of sets of traits along the slow–fast continuum. Notably, also the negative relationship between pace-of-life and thermal performance was robust against potential influences of rearing temperatures and latitude of origin, suggesting that the trade-off between a fast pace-of-life and thermal performance is general in the study species.

In summary, our study extends the recent finding of an integration between behavioural traits and thermal traits for locomotor performance [9,10] by including life history and physiology; traits typically studied under the pace-of-life syndrome hypothesis. We thereby identified a novel trade-off pattern, revealing fast-paced individuals to have lower optimum temperatures, potentially due to the energetically costly maintenance of a fast pace-of-life and/or of high optimal temperatures. We propose this trade-off to be an alternative mechanism that may contribute to the maintenance of variation in pace-of-life within populations; a topic that is still unresolved [1,4].

## Data Availability

Data supporting this manuscript are available at: https://doi.org/10.6084/m9.figshare.16910176 [65].

## References

[1] Réale D, Garant D, Humphries MM, Bergeron P, Careau V, Montiglio PO. 2010 Personality and the emergence of the pace-of-life syndrome concept at the population level. Phil. Trans. R. Soc. B **365**, 4051-4063. (10.1098/rstb.2010.0208) 21078657 PMC2992747

[2] Dammhahn M, Dingemanse NJ, Niemelä PT, Réale D. 2018 Pace-of-life syndromes: a framework for the adaptive integration of behaviour, physiology and life history. Behav. Ecol. Sociobiol. **72**, 62. (10.1007/s00265-018-2473-y)

[3] Ricklefs RE, Wikelski M. 2002 The physiology/life-history nexus. Trends Ecol. Evol. **17**, 462-468. (10.1016/S0169-5347(02)02578-8)

[4] Mathot KJ, Frankenhuis WE. 2018 Models of pace-of-life syndromes (POLS): a systematic review. Behav. Ecol. Sociobiol. **72**, 41. (10.1007/s00265-018-2459-9)

[5] Stamps JA. 2007 Growth-mortality tradeoffs and ‘personality traits’ in animals. Ecol. Lett. **10**, 355-363. (10.1111/j.1461-0248.2007.01034.x) 17498134

[6] Hämäläinen AM, Guenther A, Patrick SC, Schuett W. 2021 Environmental effects on the covariation among pace-of-life traits. Ethology **127**, 32-44. (10.1111/eth.13098)

[7] Brans KI, Stoks R, De Meester L. 2018 Urbanization drives genetic differentiation in physiology and structures the evolution of pace-of-life syndromes in the water flea *Daphnia magna*. Proc. R. Soc. B **285**, 20180169. (10.1098/rspb.2018.0169) PMC6083254 30051844

[8] Sokolova IM. 2013 Energy-limited tolerance to stress as a conceptual framework to integrate the effects of multiple stressors. Integr. Comp. Biol. **53**, 597-608. (10.1093/icb/ict028) 23615362

[9] Goulet CT, Thompson MB, Michelangeli M, Wong BBM, Chapple DG. 2017 Thermal physiology: a new dimension of the pace-of-life syndrome. J. Anim. Ecol. **86**, 1269-1280. (10.1111/1365-2656.12718) 28626934

[10] Michelangeli M, Goulet CT, Kang HS, Wong BBM, Chapple DG. 2018 Integrating thermal physiology within a syndrome: locomotion, personality and habitat selection in an ectotherm. Funct. Ecol. **32**, 970-981. (10.1111/1365-2435.13034)

[11] Sinclair BJ et al. 2016 Can we predict ectotherm responses to climate change using thermal performance curves and body temperatures? Ecol. Lett. **19**, 1372-1385. (10.1111/ele.12686) 27667778

[12] Angilletta Jr MJ. 2009 Thermal adaptation: a theoretical and empirical synthesis. Oxford, UK: Oxford University Press.

[13] Angilletta Jr MJ, Wilson RS, Navas CA, James RS. 2003 Tradeoffs and the evolution of thermal reaction norms. Trends Ecol. Evol. **18**, 234-240. (10.1016/S0169-5347(03)00087-9)

[14] Enriquez-Urzelai U, Palacio AS, Merino NM, Sacco M, Nicieza AG. 2018 Hindered and constrained: limited potential for thermal adaptation in post-metamorphic and adult *Rana temporaria* along elevational gradients. J. Evol. Biol. **31**, 1852-1862. (10.1111/jeb.13380) 30256481

[15] Richter-Boix A, Katzenberger M, Duarte H, Quintela M, Tejedo M, Laurila A. 2015 Local divergence of thermal reaction norms among amphibian populations is affected by pond temperature variation. Evolution **69**, 2210-2226. (10.1111/evo.12711) 26118477

[16] Martins F, Kruuk L, Llewelyn J, Moritz C, Phillips B. 2018 Heritability of climate-relevant traits in a rainforest skink. Heredity **122**, 41-52. (10.1038/s41437-018-0085-y) 29789644 PMC6288132

[17] Stearns SC. 1992 The evolution of life histories. Oxford, UK: Oxford University Press.

[18] Kellermann V, Chown SL, Schou MF, Aitkenhead I, Janion-Scheepers C, Clemson A, Scott MT, Sgrò CM. 2019 Comparing thermal performance curves across traits: how consistent are they? J. Exp. Biol. **222**, jeb193433. (10.1242/jeb.193433) 31085593

[19] Sgrò CM, Hoffmann AA. 2004 Genetic correlations, tradeoffs and environmental variation. Heredity **93**, 241-248. (10.1038/sj.hdy.6800532) 15280897

[20] Polverino G, Santostefano F, Díaz-Gil C, Mehner T. 2018 Ecological conditions drive pace-of-life syndromes by shaping relationships between life history, physiology and behaviour in two populations of Eastern mosquitofish. Sci. Rep. **8**, 14673. (10.1038/s41598-018-33047-0) 30279465 PMC6168454

[21] Mesas A, Jaramillo A, Castañeda LE. 2021 Experimental evolution on heat tolerance and thermal performance curves under contrasting thermal selection in *Drosophila subobscura*. J. Evol. Biol. **34**, 767-778. (10.1111/jeb.13777) 33662149

[22] Debecker S, Stoks R. 2019 Pace of life syndrome under warming and pollution: integrating life history, behavior, and physiology across latitudes. Ecol. Monogr. **89**, e01332. (10.1002/ecm.1332)

[23] Corbet PS, Suhling F, Soendgerath D. 2006 Voltinism of Odonata: a review. Int. J. Odonatol. **9**, 1-44. (10.1080/13887890.2006.9748261)

[24] Stoks R, Swillen I, De Block M. 2012 Behaviour and physiology shape the growth accelerations associated with predation risk, high temperatures and southern latitudes in *Ischnura* damselfly larvae. J. Anim. Ecol. **81**, 1034-1040. (10.1111/j.1365-2656.2012.01987.x) 22524392

[25] Brown JH, Gillooly JF, Allen AP, Savage VM, West GB. 2004 Toward a metabolic theory of ecology. Ecology **85**, 1771-1789. (10.1890/03-9000)

[26] Conover DO, Schultz ET. 1995 Phenotypic similarity and the evolutionary significance of countergradient variation. Trends Ecol. Evol. **10**, 248-252. (10.1016/S0169-5347(00)89081-3) 21237029

[27] Castañeda LE, Lardies MA, Bozinovic F. 2004 Adaptive latitudinal shifts in the thermal physiology of a terrestrial isopod. Evol. Ecol. Res. **6**, 579-593.

[28] Gaitan-Espitia JD, Bacigalupe LD, Opitz T, Lagos NA, Timmermann T, Lardies MA. 2014 Geographic variation in thermal physiological performance of the intertidal crab *Petrolisthes violaceus* along a latitudinal gradient. J. Exp. Biol. **217**, 4379-4386. (10.1242/jeb.108217) 25394627

[29] Debecker S, Sanmartín-Villar I, de Guinea-Luengo M, Cordero-Rivera A, Stoks R. 2016 Integrating the pace-of-life syndrome across species, sexes and individuals: covariation of life history and personality under pesticide exposure. J. Anim. Ecol. **85**, 726-738. (10.1111/1365-2656.12499) 26845756

[30] Boudot JP, Kalkman VJ. 2015 Atlas of the European dragonflies and damselflies. Zeist, The Netherlands: KNNV Publishing.

[31] De Block M, Pauwels K, Van Den Broeck M, De Meester L, Stoks R. 2013 Local genetic adaptation generates latitude-specific effects of warming on predator–prey interactions. Glob. Chang. Biol. **19**, 689-696. (10.1111/gcb.12089) 23504827

[32] Gilchrist GW. 1996 A quantitative genetic analysis of thermal sensitivity in the locomotor performance curve of *Aphidius ervi*. Evolution **50**, 1560-1572. (10.1111/j.1558-5646.1996.tb03928.x) 28565727

[33] Latimer CAL, McGuigan K, Wilson RS, Blows MW, Chenoweth SF. 2014 The contribution of spontaneous mutations to thermal sensitivity curve variation in *Drosophila serrata*. Evolution **68**, 1824-1837. (10.1111/evo.12392) 24576006

[34] Nakagawa S, Schielzeth H. 2010 Repeatability for Gaussian and non-Gaussian data: a practical guide for biologists. Biol. Rev. **85**, 935-956. (10.1111/j.1469-185X.2010.00141.x) 20569253

[35] Maskrey DK, Sneddon LU, Arnold KE, Wolfenden DCC, Thomson JS. 2020 The impact of personality, morphotype and shore height on temperature-mediated behavioural responses in the beadlet anemone *Actinia equina*. J. Anim. Ecol. **89**, 2311-2324. (10.1111/1365-2656.13301) 32830317

[36] Padfield D, O'Sullivan H, Pawar S. 2021 rTPC and nls.multstart: a new pipeline to fit thermal performance curves in R. Methods Ecol. Evol. **12**, 1138-1143. (10.1111/2041-210X.13585)

[37] Grace JB, Michael Anderson T, Han O, Scheiner SM. 2010 On the specification of structural equation models for ecological systems. Ecol. Monogr. **80**, 67-87. (10.1890/09-0464.1)

[38] Dingemanse NJ, Dochtermann N, Wright J. 2010 A method for exploring the structure of behavioural syndromes to allow formal comparison within and between data sets. Anim. Behav. **79**, 439-450. (10.1016/j.anbehav.2009.11.024)

[39] Mugel SG, Naug D. 2020 Metabolic rate shapes phenotypic covariance among physiological, behavioral, and life-history traits in honeybees. Behav. Ecol. Sociobiol. **74**, 129. (10.1007/s00265-020-02901-5)

[40] Kline RB. 2016 *Principles and practice of structural equation modeling*, 4th edn. New York, NY: Guilford Press.

[41] R Core Team. 2021 R: a language and environment for statistical computing. Vienna, Austria: R Foundation for Statistical Computing.

[42] Bates D, Mächler M, Bolker B, Walker S. 2015 Fitting linear mixed-effects models using lme4. J. Stat. Softw. **67**, 1-48. (10.18637/jss.v067.i01)

[43] Lenth R. 2021 emmeans: Estimated marginal means, aka least-squares means. R Package. version 1.6.3.

[44] Rosseel Y. 2012 lavaan: An R Package for structural equation modeling. J. Stat. Softw. **48**, 1-36. (10.18637/JSS.V048.I02)

[45] Wikelski M, Spinney L, Schelsky W, Scheuerlein A, Gwinner E. 2003 Slow pace of life in tropical sedentary birds: a common-garden experiment on four stonechat populations from different latitudes. Proc. R. Soc. B **270**, 2383-2388. (10.1098/rspb.2003.2500) PMC1691521 14667355

[46] Buckley LB, Nufio CR, Kirk EM, Kingsolver JG. 2015 Elevational differences in developmental plasticity determine phenological responses of grasshoppers to recent climate warming. Proc. R. Soc. B **282**, 20150441. (10.1098/rspb.2015.0441) PMC4590449 26041342

[47] Huey RB, Kingsolver JG. 1989 Evolution of thermal sensitivity of ectotherm performance. Trends Ecol. Evol. **4**, 131-135. (10.1016/0169-5347(89)90211-5) 21227334

[48] Yamahira K, Conover DO. 2002 Intra- vs. interspecific latitudinal variation in growth: adaptation to temperature or seasonality? Ecology **83**, 1252-1262. (10.1890/0012-9658(2002)083[1252:IVILVI]2.0.CO;2)

[49] Tüzün N, de Beeck LO, Brans KI, Janssens L, Stoks R. 2017 Microgeographic differentiation in thermal performance curves between rural and urban populations of an aquatic insect. Evol. Appl. **10**, 1067-1075. (10.1111/eva.12512) 29151861 PMC5680628

[50] Klepsatel P, Gáliková M, De Maio N, Huber CD, Schlötterer C, Flatt T. 2013 Variation in thermal performance and reaction norms among populations of *Drosophila melanogaster*. Evolution **67**, 3573-3587. (10.1111/evo.12221) 24299409

[51] Jupe LL, Bilton DT, Knights AM. 2020 Do differences in developmental mode shape the potential for local adaptation? Ecology **101**, e02942. (10.1002/ECY.2942) 31778204

[52] Tüzün N, Stoks R. 2018 Evolution of geographic variation in thermal performance curves in the face of climate change and implications for biotic interactions. Curr. Opin. Insect Sci. **29**, 78-84. (10.1016/j.cois.2018.07.004) 30551830

[53] Johansson F, Stoks R, Rowe L, De Block M. 2001 Life history plasticity in a damselfly: effects of combined time and biotic constraints. Ecology **82**, 1857-1869. (10.2307/2680052)

[54] Mathot KJ, Dingemanse NJ, Nakagawa S. 2019 The covariance between metabolic rate and behaviour varies across behaviours and thermal types: meta-analytic insights. Biol. Rev. **94**, 1056-1074. (10.1111/brv.12491) 30588731

[55] Laskowski KL, Moiron M, Niemelä P. 2020 Integrating behavior in life-history theory: allocation versus acquisition? Trends Ecol. Evol. **36**, 132-138. (10.1016/j.tree.2020.10.017) 33203522

[56] Tüzün N, Müller S, Koch K, Stoks R. 2017 Pesticide-induced changes in personality depend on the urbanization level. Anim. Behav. **134**, 45-55. (10.1016/j.anbehav.2017.10.007)

[57] Huey RB, Kearney MR, Krockenberger A, Holtum JAM, Jess M, Williams SE. 2012 Predicting organismal vulnerability to climate warming: roles of behaviour, physiology and adaptation. Phil. Trans. R. Soc. B **367**, 1665-1679. (10.1098/rstb.2012.0005) 22566674 PMC3350654

[58] Roze T, Christen F, Amerand A, Claireaux G. 2013 Trade-off between thermal sensitivity, hypoxia tolerance and growth in fish. J. Therm. Biol. **38**, 98-106. (10.1016/J.JTHERBIO.2012.12.001)

[59] Feder ME, Hofmann GE. 1999 Heat-shock proteins, molecular chaperones, and the stress response: evolutionary and ecological physiology. Annu. Rev. Physiol. **61**, 243-282. (10.1146/annurev.physiol.61.1.243) 10099689

[60] Sørensen JG, Kristensen TN, Loeschcke V. 2003 The evolutionary and ecological role of heat shock proteins. Ecol. Lett. **6**, 1025-1037. (10.1046/j.1461-0248.2003.00528.x)

[61] Stoks R, De Block M. 2011 Rapid growth reduces cold resistance: evidence from latitudinal variation in growth rate, cold resistance and stress proteins. PLoS ONE **6**, e16935. (10.1371/journal.pone.0016935) 21390210 PMC3044720

[62] Enders EC, Wall AJ, Svendsen JC. 2019 Hypoxia but not shy-bold phenotype mediates thermal preferences in a threatened freshwater fish, *Notropis percobromus*. J. Therm. Biol. **84**, 479-487. (10.1016/J.JTHERBIO.2019.08.001) 31466789

[63] Cerqueira M, Rey S, Silva T, Featherstone Z, Crumlish M, MacKenzie S. 2016 Thermal preference predicts animal personality in Nile tilapia *Oreochromis niloticus*. J. Anim. Ecol. **85**, 1389-1400. (10.1111/1365-2656.12555) 27219014

[64] Peiman KS, Robinson BW. 2017 Comparative analyses of phenotypic trait covariation within and among populations. Am. Nat. **190**, 451-468. (10.1086/693482) 28937814

[65] Tüzün N, Stoks R. 2022 Data from: A fast pace-of-life is traded off against a high thermal performance. *Figshare*. (10.6084/m9.figshare.16910176) PMC9006028 35414235

